# Inhibition of DNA methyltransferase as a novel therapeutic strategy to overcome acquired resistance to dual PI3K/mTOR inhibitors

**DOI:** 10.18632/oncotarget.3016

**Published:** 2015-02-11

**Authors:** Xiao-jun Qian, Yun-tian Li, Yan Yu, Fen Yang, Rong Deng, Jiao Ji, Lin Jiao, Xuan Li, Rui-Yan Wu, Wen-Dan Chen, Gong-Kan Feng, Xiao-Feng Zhu

**Affiliations:** ^1^ State Key Laboratory of Oncology in South China, Collaborative Innovation Center for Cancer Medicine, Cancer Center, Sun Yat-sen University, Guangzhou 510060, China; ^2^ Department of Oncology, Anhui Provincial Hospital, Affiliated to Anhui Medical University, Hefei 230001, China; ^3^ Department of Biochemistry and Molecular Biology, Nanjing Medical University, Nanjing 210000, China

**Keywords:** BEZ235, Acquired Resistance, DNA Methyltransferase, Nasopharyngeal Carcinoma

## Abstract

Dual PI3K/mTOR(phosphatidylinositol 3-kinase/mammalian target of rapamycin) inhibitors are being evaluated clinically for the treatment of tumors with a hyperactivated PI3K/mTOR pathway. However, unexpected outcomes were obtained in clinical studies of cancer patients with an aberrant PI3K pathway. In clinical trials, applicable combination regimens are not yet available. In this study, using an integrated analysis of acquired BEZ235-resistant nasopharyngeal carcinoma cells, we demonstrate that DNA methyltransferase is a key modulator and a common node upstream of the AKT/mTOR and PDK1/MYC pathways, which are activated in cancer cells with acquired BEZ235 resistance. DNA methyltransferases were upregulated and induced PTEN and PPP2R2B gene hypermethylation, which downregulated their expression in BEZ235-resistant cancer cells. Reduced PTEN and PPP2R2B expression correlated with activated AKT/mTOR and PDK1/MYC pathways and conferred considerable BEZ235 resistance in nasopharyngeal carcinoma. Targeting methyltransferases in combination with BEZ235 sensitized BEZ235-resistant cells to BEZ235 *in vitro* and *in vivo*, suggesting the potential clinical application of this strategy to overcome BEZ235 resistance.

## INTRODUCTION

Targeted therapies might represent the future of personalized medicine for cancer patients. However, the phosphatidylinositol 3-kinase (PI3K)-AKT pathway is one of the most commonly deregulated signaling pathways in human cancers [1]. Genetic aberrations that affect this pathway, such as mutations that activate PIK3CA or inactivate PTEN, have been identified in virtually all epithelial tumors [2]. It has been reported that BEZ235, one of the dual PI3K/mTOR(phosphatidylinositol 3-kinase/mammalian target of rapamycin) inhibitors, has a potent inhibitory effect in numerous tumors such as breast cancer [3], hepatic carcinoma [4], ovarian carcinoma [5], prostate cancer [6], medulloblastoma [7], non-small cell lung cancer [8, 9], non-Hodgkin lymphoma [10, 11], liposarcoma [12], cholangiocarcinoma [13], leukemia [14], pancreatic cancer [15, 16] and nasopharyngeal carcinoma [17] in preclinical studies, especially in PIK3CA mutant subtypes. PIK3CA gene mutation and amplification are observed frequently in NPC, especially in advanced stages of disease [18], indicating a potential therapeutic effect of the dual PI3K/mTOR inhibitor in nasopharyngeal carcinoma. BEZ235 significantly inhibits cell proliferation in nasopharyngeal carcinoma both *in vitro* and *in vivo* by blocking the PI3K/AKT/mTOR pathway [17, 19].

However, the outcomes of BEZ235 for phase I and phase II trials are unsatisfactory. Exploring the mechanism(s) underlying these unexpected outcomes, researchers have found that primary and acquired resistance could be involved. The former might occur in cancers without hyperactivation of the PI3K/AKT/mTOR pathway or activation of alternative pathways. The latter might result from the mutation of targets or activation of alternative pathways that lead to ineffective clinical therapies. Although multiple mechanisms of BEZ235 resistance have been identified in preclinical studies, the underlying mechanisms are varied in different tumors. In addition, the acquired resistance to BEZ235 remains elusive. Elucidation of the mechanism underlying acquired resistance will contribute to the design of anticancer treatment strategies. For example, blockade of the PI3K pathway activates AR signaling in prostate cancer and results in elevated pHER3 in breast cancer, and combination therapies have been shown to effectively improve cancer regression [20, 21].

In this study, we report a rationally designed therapy to conquer BEZ235 resistance that may benefit patients with aberrant PI3K/mTOR pathway-associated nasopharyngeal carcinoma. We found that the two survival signaling pathways, AKT/mTOR and PDK1/MYC, were activated in cells with acquired BEZ235 resistance. Next, we identified DNA methyltransferases as a common node that is overexpressed in resistant models. We also showed direct activation of the AKT/mTOR and PDK1/MYC pathways by PTEN and PPP2R2B methylation, which is induced by overexpression of DNA methyltransferases. Notably, targeting this key node with a DNA methyltransferase inhibitor universally sensitized resistant cells to BEZ235 treatment *in vitro* and *in vivo*. Our data indicate that combination treatment with a methyltransferase inhibitor together with BEZ235 could be a useful strategy to overcome BEZ235 resistance.

## RESULTS

### DNA hypermethylation in acquired dual PI3K/mTOR inhibitor-resistant cells

**Figure 1:**
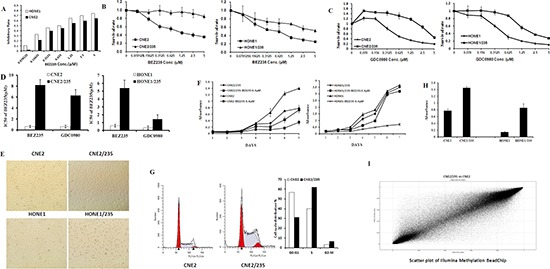
DNA hypermethylation in acquired dual PI3K/mTOR inhibitors resistant cells **(A)** Inhibitory effects of BEZ235 on CNE2 and HONE1 cell proliferation. Cell growth was assessed using the MTT assay after treatment with BEZ235 for 5 d. **(B)** Effect of BEZ235 on cell proliferation in the parental nasopharyngeal carcinoma cell lines and their corresponding acquired BEZ235 sublines using the MTT assay. **(C)** Effect of GDC0980 on cell proliferation in the parental nasopharyngeal carcinoma cell lines and their corresponding acquired BEZ235 sublines using the MTT assay. **( D)** IC50 values of BEZ235 in parental cell lines and their corresponding resistant cells. The data shown are representative of 3 individual experiments. **(E)** Representative microscopic images of the parental cell lines CNE2 and HONE1 and their resistant CNE2/235 and HONE1/235 cells grown in 6-well plates. **(F)** Growth curves were calculated for 7 d using the MTT assay with or without 0.4 μM BEZ235. **(G)** The cell cycle was analyzed in parental cell lines and resistant cell lines by PI staining and analyzed by flow cytometry. **(H)** Parental cells and their resistant counterparts were plated in matrigel-coated 96-well plates. Adhesion was analyzed using the MTT assay. (*p < 0.05; **p < 0.01). **(I)** Gene methylation was determined using Illumina Methylation BeasChip assays in the CNE2 and CNE2/235 cell lines. Dots on the bit line indicate no difference between CNE2 and CNE2/235.

We modeled the development of acquired resistance in patients by treating PIK3CA mutant nasopharyngeal carcinoma cells (CNE2, HONE1) with increasing doses of BEZ235 for 6 to 9 months to select BEZ235-resistant sublines (CNE2/235, HONE1/235) (Supplementary Table S1). The resistant sublines were cultured in 0.4 μmol/L BEZ235. The concentration was chosen because it induced 50% apoptotic cells in the parental lines (Figure 1A). Compared with the parental lines, the resistant cells were significantly more resistant (9.0–13.2 fold) to BEZ235 treatment *in vitro* (Figures 1B and 1D). Interestingly, the BEZ235 sublines with acquired resistance were also resistant to GDC0980 (4.2–10.5 fold), another dual PI3K/mTOR inhibitor that differs structurally from BEZ235 (Figures 1C and 1D). The resistant cells were able to form stable populations and could be passaged in the presence of BEZ235 (Figure 1E). Despite resistance to apoptosis, the BEZ235-selected cells proliferated significantly slower than their parental counterparts (Figure 1F). In addition, cell cycle analysis by flow cytometry showed that the resistant cells were arrested at the S/G2 transition, indicating the mechanism underlying the slow growth (Figure 1G). A basement membrane model was used to evaluate the adhesion activity of the cells. Our results indicated that cell adhesion was significantly promoted in resistant cells (Figure 1H).

As noted, the most common mechanism of resistance to kinase inhibitors involves sustained activation of the downstream and bypass signaling pathways. In explorations of the mechanism of signaling pathway activation, overexpression of receptor tyrosine kinases (RTKs) is the best understood mechanism [22]. However, compared to parental cells, overexpression of RTKs (data not shown) has not been observed in resistant sublines. To further explore the mechanism of resistance, whole gene methylation analysis using an Illumina Methylation BeasChip was performed in the sensitive parental cells and the resistant cells. The 37395 CpG probes were found to be differentially methylated; two-thirds were hypermethylated in resistant cells (Figure 1I). Promoter methylation and negative expression of tumor suppressor genes have been shown to be involved in tumorigenesis. Compared to parental cells, BEZ235-resistant cells displayed promoter hypermethylation of the tumor suppressor genes PTEN and PPP2R2B(Supplementary Table S2), suggesting that methylation of these genes may function to promote cancer proliferation.

### PTEN hypermethylation activates the PI3K/mTOR signaling pathway to induce BEZ235 resistance

**Figure 2:**
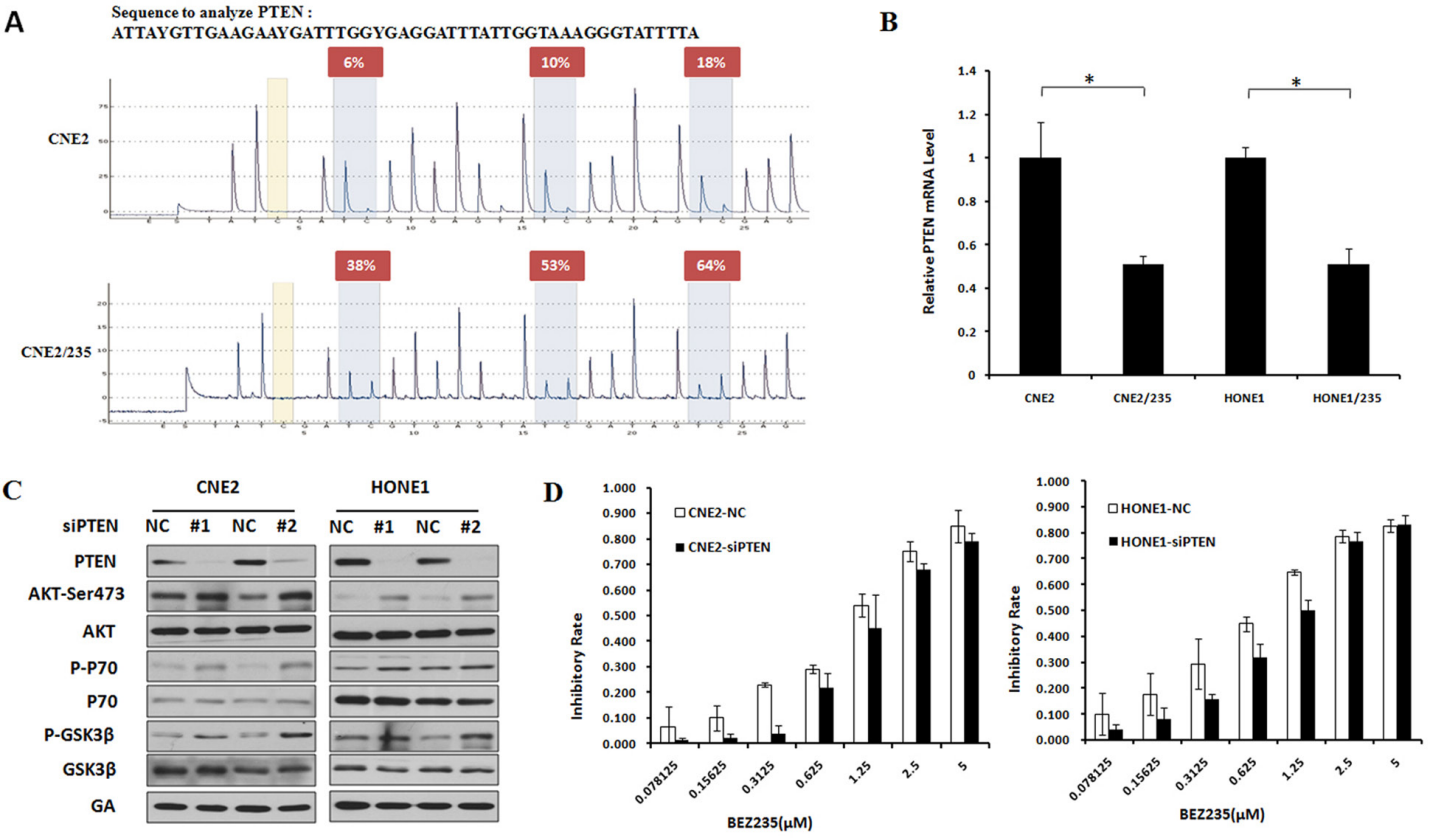
PTEN hypermethylation activates the PI3K/mTOR signaling pathway to induce BEZ235 resistance **(A)** PTEN CpG island methylation was determined by pyrosequencing assays in CNE2 and CNE2/235 cell lines. **(B)** PTEN mRNA levels were examined using a quantitative PCR assay in parental and resistant cell lines. **(C)** Effect of PTEN knockdown by siRNA on AKT, GSK3β and P70 phosphorylation in CNE2 and HONE1 cell lines by immunoblotting analysis. **(D)** Sensitivity to BEZ235 after PTEN knockdown by siRNA in CNE2 and HONE1 cell lines.

PTEN is a negative regulator of the PI3K/AKT/mTOR pathway, and PTEN promoter methylation is an alternative mechanism of PTEN deletion [23]. PTEN promoter methylation correlates with decreased PTEN protein expression, which often increases AKT/mTOR pathway activation in tumor progression [24]. To verify the gene hypermethylation reported using the Methylation Chip, a pyrosequencing technique was conducted to analyze the methylation status of PTEN promoters. The PTEN promoters analyzed by pyrosequencing were selected based on the Methylation Chip findings. Compared to parental cells, PTEN promoter methylation clearly increased (Figure 2A). We next investigated whether PTEN promoter methylation correlated with PTEN protein expression. PTEN expression was reduced in two resistant cell lines, suggesting an inverse association between PTEN promoter methylation and PTEN protein expression (Figure 2B). The loss of PTEN expression in parental cells suggested that it might contribute to BEZ235 resistance. To investigate this possibility, we created a PTEN deficiency cells in the parental sublines by PTEN knockdown with siRNA. PTEN knockdown increased the phosphorylation of AKT, P70 and GSK3β, which are known key proteins in the PI3K/AKT/mTOR pathway (Figure 2C). PTEN knockdown universally induced resistance of nasopharyngeal carcinoma cells to BEZ235 treatment (Figure 2D). These data indicate that PTEN deficiency induced by hypermethylation activates the PI3K/AKT/mTOR pathway, which is involved in BEZ235 resistance.

### PPP2R2B hypermethylation modulates MYC and P70 phosphorylation to induce BEZ235 resistance

**Figure 3:**
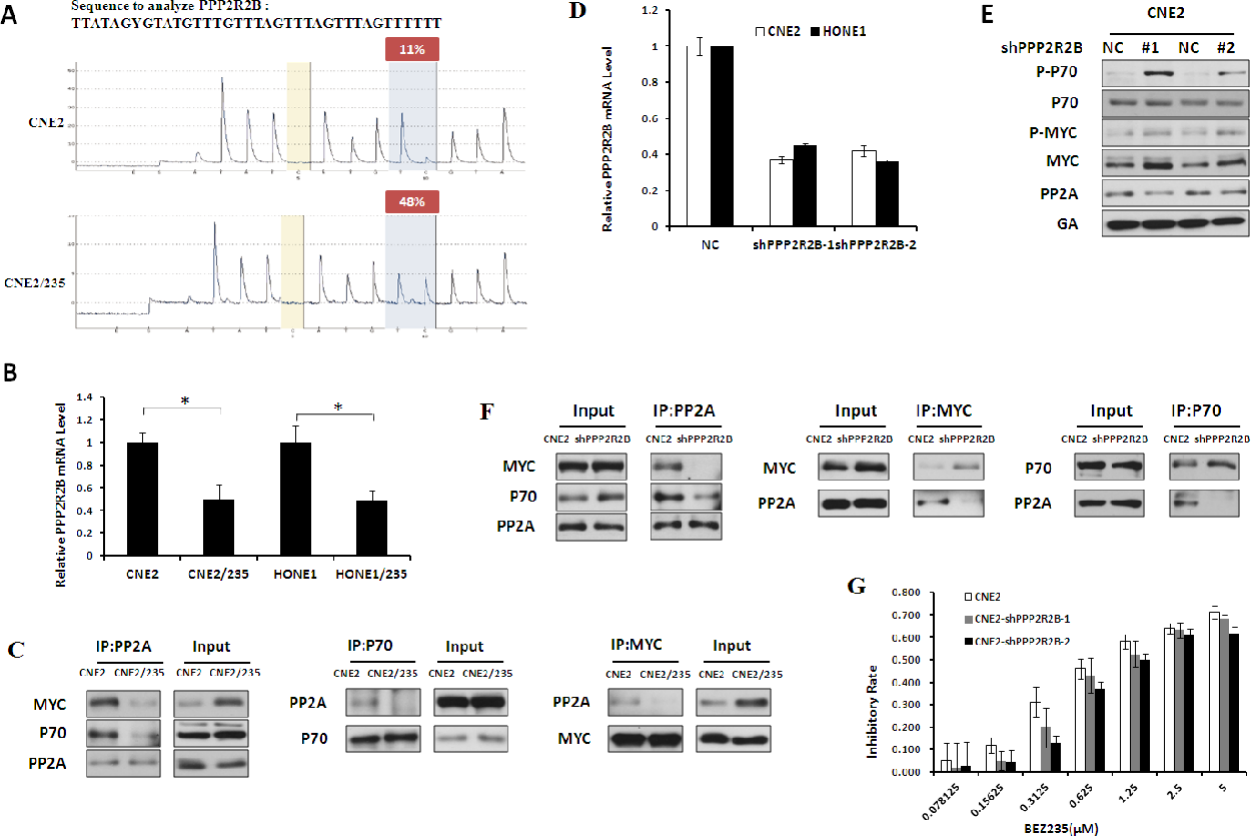
PPP2R2B hypermethylation modulates MYC and P70 phosphorylation to induce BEZ235 resistance **(A)** PPP2R2B CpG island methylation was determined in CNE2 and CNE2/235 cell lines using a pyrosequencing assay. **(B)** The levels of PPP2R2B mRNA were determined using a quantitative PCR assay in parental and resistant cell lines. **(C)** The interactions between MYC and PP2A and between P70 and PP2A were detected by immunoprecipitation in CNE2 and CNE2/235 cell lines. **(D)** The levels of PPP2R2B mRNA were examined by q-PCR after transfection with PPP2R2B shRNA in CNE2 and HONE1. **(E)** Effect of PPP2R2B knockdown with shRNA on MYC and P70 phosphorylation in CNE2 cell line. **(F)** Effect of PPP2R2B knockdown on the interactions between MYC and PP2A and between P70 and PP2A in the CNE2 cell line based on immunoprecipitation. **(G)** The sensitivity of CNE2 to BEZ235 after PPP2R2B knockdown by PPP2R2B shRNA using the MTT assay.

Protein phosphatase 2A (PP2A) functions as a multimeric enzyme that contains the catalytic C subunit, a scaffolding A subunit and a regulatory B subunit [25]. Various B regulatory subunits confer substrate specificity for dephosphorylation events conducted by the C subunit [26]. P70 and MYC have been previously identified to be substrates of PP2A with the same B subunit called B55β, which is encoded by the PPP2R2B gene [27]. Our results showed that PPP2R2B promoter methylation correlated with decreased PPP2R2B expression (Figure 3A and 3B). To dissect the possible mechanisms responsible for hyperactivation of MYC and P70 by PPP2R2B hypermethylation, immunoprecipitation assays were performed. The results showed that the interaction of PP2A (C subunit) with both MYC and P70 increased in resistant cells (Figure 3C and S1A). We speculate that a PPP2R2B deficiency can induce MYC and P70 phosphorylation by impairing the binding of PP2A and MYC as well as PP2A and P70, thus modulating nasopharyngeal carcinoma resistance to BEZ235. To investigate this hypothesis, PPP2R2B-deficient cells were established in parental cells by knockdown of PPP2R2B. Compared to the negative control, MYC and P70 phosphorylation evens were significantly increased in response to the knockdown of PPP2R2B (Figure 3D, and S1B). Moreover, immunoprecipitation assays demonstrated a reduction of the direct interaction between PP2A and MYC and between PP2A and P70 in response to PPP2R2B knockdown (Figure 3F and S1C). Furthermore, as we expected, PPP2R2B knockdown attenuated BEZ235-induced growth inhibition (Figure 3G and S1D). Taken together, silencing of PPP2R2B by methylation correlated with MYC and P70 hyperphosphorylation and BEZ235 resistance.

### Activation of the AKT/mTOR and PDK1/MYC pathways drives resistance in cells with acquired BEZ235 resistance

**Figure 4:**
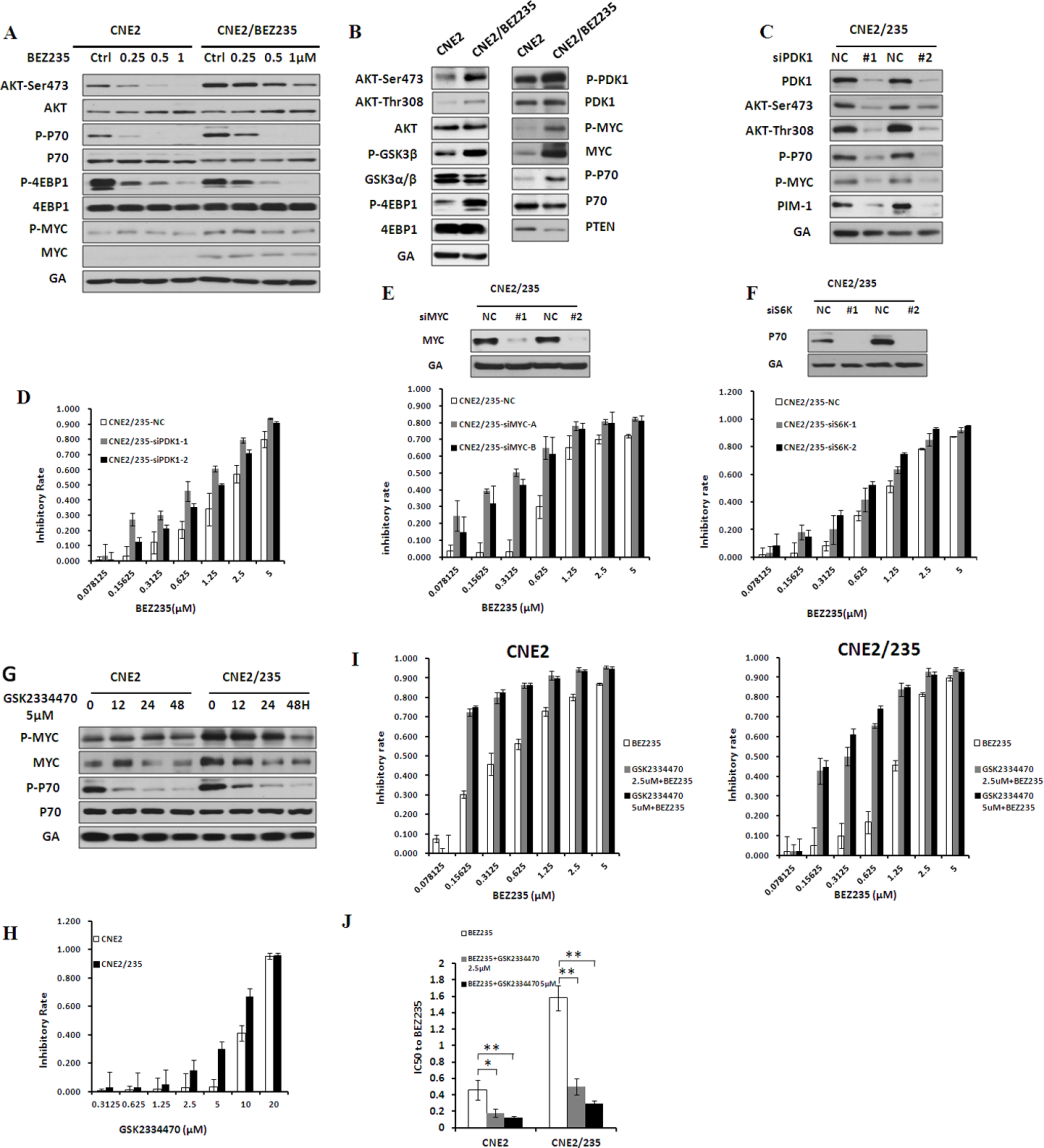
Activation of PI3K/AKT/mTOR and PDK1/MYC survival pathway in BEZ235-resistant cells **(A)** Effect of BEZ235 on the PI3K/AKT/mTOR and PDK1/MYC survival pathway in CNE2 and CNE2/235 cells by immunoblotting analysis. **(B)** Differential activation of the PI3K/AKT/mTOR and PDK1/MYC survival pathways in CNE2 and CNE2/235 cells by immunoblotting analysis. **(C)** Effect of PDK1 knockdown on P-AKT, P-P70, P-Myc, and PIM1 expression in CNE2/235 cells. **(D)** Sensitivity to BEZ235 in CNE2/235 cells after PDK1 knockdown using the MTT assay. **(E)** Effect of MYC knockdown on the sensitivity to BEZ235 in CNE2/235 cells. **(F)** Effect of P70(S6K) knockdown on the sensitivity to BEZ235 in CNE2/235 cells. **(G)** Effect of the PDK1 inhibitor GSK2334470 on MYC and P70 phosphorylation in CNE2/235 cell line by immunoblotting analysis. **(H)** Sensitivity to GSK2334470 in CNE2/235 cells after treatment with GSK2334470 for 5 d using the MTT assay. **(I)** Inhibitory effect of GSK2334470 in combination with BEZ235 on cell proliferation in CNE2/235 cells using the MTT assay. **(J)** IC50 values of BEZ235 with or without GSK2334470 in CNE2 and CNE2/235 cell lines. The data shown are representative of 3 individual experiments.

We treated parental and resistant cells with BEZ235 simultaneously. The results showed that inhibition of the AKT/mTOR pathway by BEZ235 was attenuated in resistant cells (Figure 4A and S2A). In fact, we found a substantial upregulation of AKT/mTOR and PDK1/MYC pathway activity in resistant sublines compared with parental cell lines (Figure 4B and S2B), suggesting that increased AKT/mTOR and PDK1/MYC signaling may be acquired resistance mechanisms. To determine whether AKT/mTOR and PDK1/MYC signaling activation is sufficient to confer BEZ235 resistance, we modulated key proteins in the two survival signaling pathways, such as PDK1, MYC and P70, using siRNA in resistant cells. PDK1 knockdown inhibited the phosphorylation of AKT, P70 and MYC as well as PIM-1 protein expression in resistant cells (Figure 4C and S2C). PDK1 knockdown universally sensitized BEZ235-resistant cells to BEZ235 treatment (Figure 4D and S2D). Next, we examined whether siMYC and siP70 had a similar BEZ235 sensitization effect in BEZ235-resistant cells. Compared with the negative control, MYC and P70 knockdown by siRNA sensitized cells to BEZ235 treatment in two resistant sublines (Figures 4E, 4F, S2E, S2F). These data indicate an essential role of AKT/mTOR and PDK1/MYC signaling activation in mediating acquired BEZ235 resistance in nasopharyngeal carcinoma.

The anticancer activity of the PDK1 inhibitor GSK2334470 has been reported [28, 29]. In resistant cells, GSK2334470 significantly induced a time-dependent inhibition of MYC and P70 phosphorylation (Figure 4G and S2G). The resistance mechanism induced by PDK1 activation prompted us to investigate the possibility that GSK2334470 together with BEZ235 reverses BEZ235 resistance. Thus, we compared the effect of GSK2334470 on the cell viability of resistant cells as well as their parental counterparts. The data showed that GSK2334470-induced growth inhibition was much more effective in resistant cells (Figure 4H and S2H). We examined whether GSK2334470 could sensitize BEZ235-resistant cells to the BEZ235 treatment. Indeed, the combination treatment considerably increased BEZ235-mediated growth inhibition, especially in resistant cells (Figures 4I, 4J, S2I, S2J). Together, these data indicate that PDK1 activation and their downstream signaling events (MYC and P70 activation) are involved in BEZ235 resistance, which can be effectively blocked with a PDK inhibitor. PDK1 inhibition by GSK2334470 sensitizes BEZ235-resistant cells to BEZ235.

### PIM1 induces MYC phosphorylation to drive BEZ235 resistance

**Figure 5:**
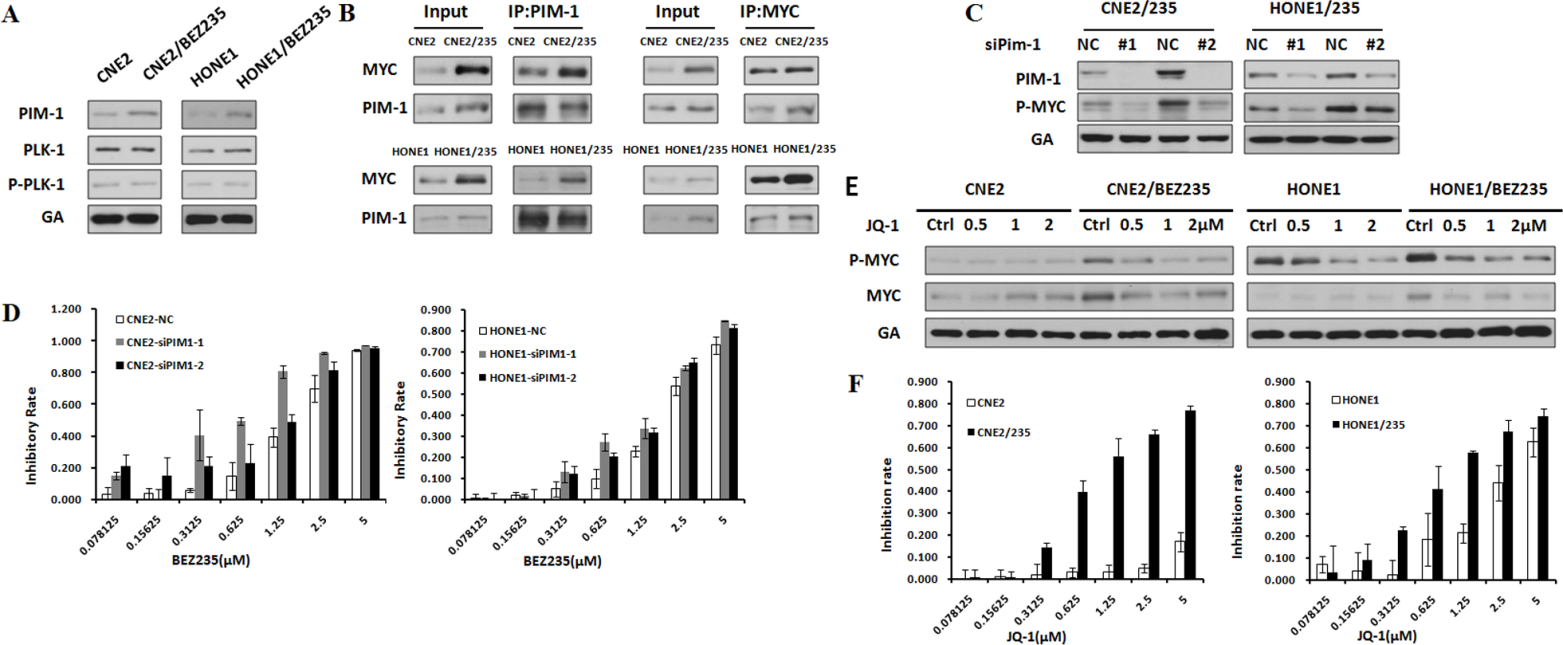
PIM1 Induces MYC Phosphorylation that Drivers BEZ235 Resistance **(A)** Differential expression of PIM1, P-PLK, and PLK proteins in the parental and BEZ235-resistant cells by immunoblotting analysis. **(B)** The interaction between MYC and PIM1 was detected using an immunoprecipitation assay in parental and resistant cell lines. **(C)** Effect of PIM1 knockdown on P-Myc expression in CNE2/235 and HONE1/235 cells. **(D)** Sensitivity to BEZ235 in BEZ235-resistant cells after PIM1 knockdown using the MTT assay. **(E)** Effect of the MYC inhibitor JQ-1 on MYC phosphorylation in BEZ235-resistant cell lines. **(F)** Sensitivity of BEZ235-resistant cells to JQ-1 using the MTT assay.

To investigate a kinase downstream of PDK1 that is responsible for MYC induction, we evaluated the expression of PIM1 and PLK1, which has been reported to induce MYC phosphorylation [30–33]. Compared with parental lines, PIM1 but not PLK1 was overexpressed in the resistant cells (Figure 5A) and could be suppressed by siPDK1 (Figure 4C). PIM1 stabilizes MYC by mediating MYC phosphorylation, thus enhancing the transcriptional activity of c-MYC and contributing to tumorigenesis. We next tested the possibility that PIM1 interacts directly with MYC. The results of the immunoprecipitation analysis showed that PIM1 interacted directly with MYC in resistant cells (Figure 5B). To elucidate the mechanism by which PIM1 induced MYC phosphorylation in resistant cells, we assessed MYC phosphorylation in PIM1 knockdown cells by siRNA. PIM1 knockdown substantially reduced MYC phosphorylation and restored the sensitivity of these cells to BEZ235 treatment (Figures 5C and 5D). These data indicate that PIM1 directly and specifically phosphorylates MYC and thus confers BEZ235 resistance.

MYC is a master regulatory factor in BEZ235 resistance. Nevertheless, a therapeutic approach to target MYC has remained elusive [27]. The absence of a clear ligand-binding domain has posed a formidable obstacle toward direct inhibition [34]. Therefore, researchers have developed the BRD4 inhibitor JQ-1 to target the transcriptional functions of MYC by interfering with BRD4-induced MYC transcriptional initiation and elongation [35–37]. In resistant cells, JQ-1 induced a significant dose-dependent inhibition of MYC phosphorylation (Figure 5E). Thus, we compared the effect of JQ-1 on the cell viability of resistant cells as well as their parental counterparts. The data showed that JQ-1-induced growth inhibition was much more effective in resistant cells than in parental cells that were not resistant (Figure 5F). These data indicate that PIM1-mediated MYC activation is critical in acquired BEZ235 resistance and that inhibition of MYC effectively inhibits the growth of resistant cells.

### Inhibition of DNA methyltransferase activity reverses BEZ235 resistance

**Figure 6:**
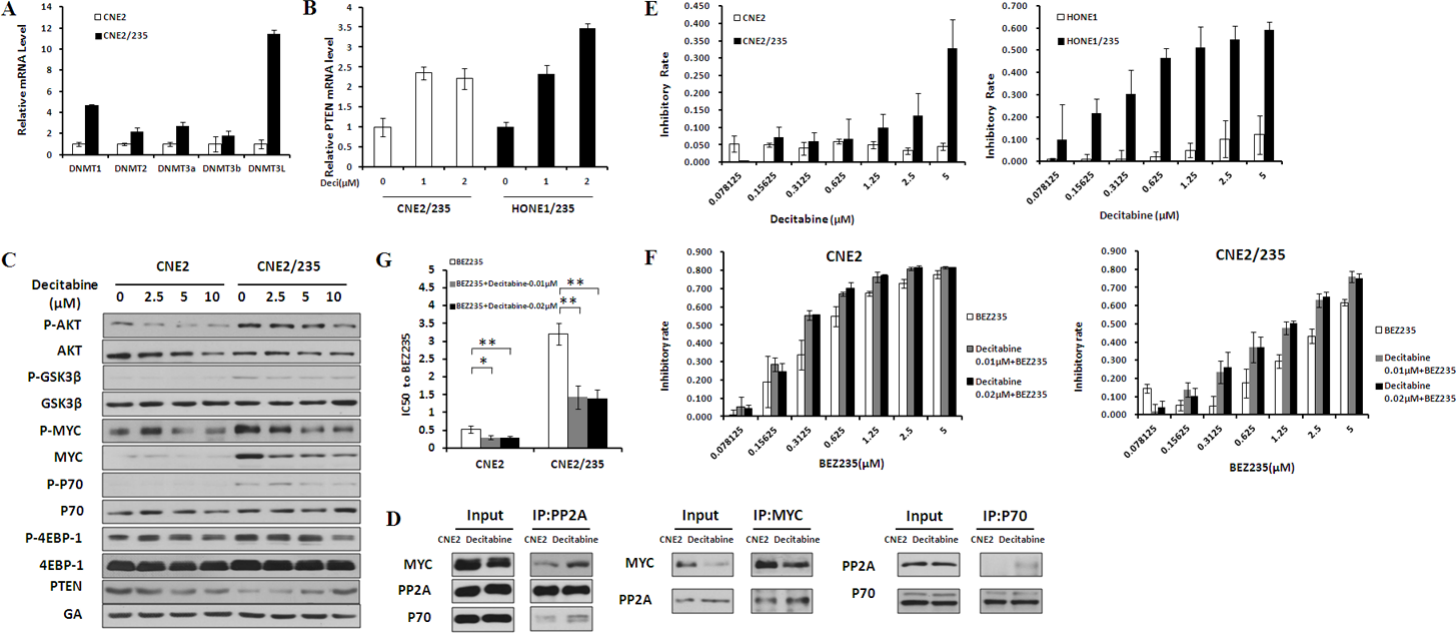
Inhibition of DNA Methyltransferase activity reverses BEZ235 resistance **(A)** The mRNA levels of DNA methyltransferases were analyzed in CNE2 and CNE2/235 cell lines by q-PCR. **(B)** PPP2R2B and PTEN mRNA levels were analyzed in resistant cell lines after treatment with the DNA methylation inhibitor decitabine at the indicated concentrations for 72 h. **(C)** Effects of decitabine on the expression of survival pathway proteins at the indicated concentrations. **(D)** Effects of decitabine on the binding of MYC and PP2A and of P70 and PP2A in the CNE2/235 cell line. Cells were treated with 1 μM decitabine for 48 h. Immunoprecipitation was conducted. **(E)** Effect of the DNA methylation inhibitor decitabine on cell proliferation in the parental and resistant cell lines using the MTT assay. **(F)** Inhibitory effect of decitabine and BEZ235 on cell proliferation in CNE2 and CNE2/235 cell lines. Cells were treated for 5 d at the indicated concentrations. The MTT assay was conducted. **(G)** The IC50 values of BEZ235 with or without decitabine were determined in CNE2 and CNE2/235 cell lines using the MTT assay. The data shown are representative of 3 individual experiments.

DNA methylation is a classic epigenetic modification in mammalian cells that involves the addition of a methyl group to cytosine generally at the CpG dinucleotide by DNA methyltransferases (Dnmts), resulting in 5-methylcytosine (meC) [38]. The mammalian DNMT family includes four active members: DNMT1, DNMT2, DNMT3A, DNMT3B, and DNMT3L. Mammalian DNMTs are responsible for methylation pattern acquisition during tumor development [39]. Human DNMTs were coordinately upregulated at the level of mRNA expression in BEZ235-resistant cells (Figure 6A and S3A). Inhibition of DNMTs correlates with a reduced tumorigenicity and increased expression of tumor suppressor genes. The most commonly used inhibitor, decitabine, is effective against several cancers by blocking DNA methylation [40]. We next examined whether decitabine is involved in PTEN and PPP2R2B expression and thus in BEZ235 resistance. The mRNA levels of the two genes were elevated in a concentration-dependent manner following treatment with decitabine (Figure 6B). Furthermore, decitabine dephosphorylated key proteins in the AKT/mTOR and PDK1/MYC pathways, such as AKT, GSK3β, MYC, P70, and 4EBP-1, and upregulated PTEN protein expression (Figure 6C and S3B). Moreover, immunoprecipitation analysis demonstrated that the interaction between MYC and PP2A and between P70 and PP2A were increased (Figure 6D and S3C). These data indicate that inhibition of methyltransferases enhances PTEN and PPP2R2B gene expression and attenuates the AKT/mTOR and PDK1/MYC pathways. Decitabine treatment clearly induced growth inhibition in resistant cells (Figure 6E), and the phenomenon can also be found in an alternative methyltransferases inhibitor RG108 (Figure S4A). In addition, methyltransferases inhibitors provided a more effective sensitization of BEZ235-resistant cells to BEZ235 treatment compared with the parental cells (Figure 6F, 6G, S3D, S3E, S4B and S4C). These consistent data demonstrate that inhibition of DNA methyltransferases can reverse BEZ235 resistance.

### Targeting DNA methyltransferases overcomes BEZ235 resistance *in vivo*

**Figure 7:**
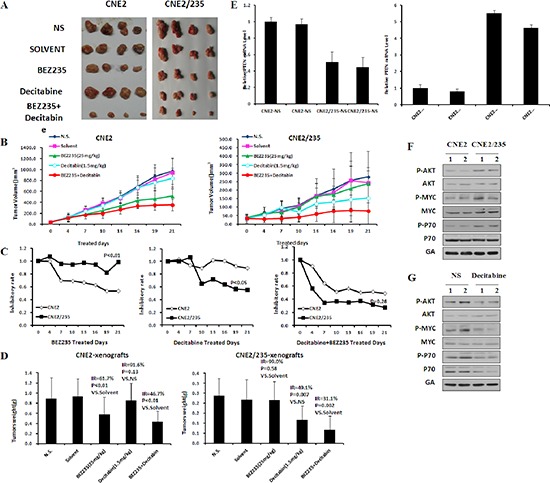
Targeting methyltransferase overcomes BEZ235 resistance *in vivo* **(A) (B)** Xenograft tumor growth of CNE2 and CNE2/235 cells in nude mice treated with BEZ235 at 25 mg/kg per day or decitabine 1.5 mg/kg three times per week. (*p* values in CNE2: *p* = 0.27 in Decitabine vs slovent, *p* < 0.01 in BEZ235 vs NS, *p* < 0.01 in combination vs solvent, *p* = 0.55 in combination vs BEZ235; *p* values in CNE2/235: *p* = 0.90 in Decitabine vs slovent, *p* = 0.03 in BEZ235 vs NS, *p* < 0.01 in combination vs solvent, *p* = 0.03 in combination vs Decitabine) **(C)** The inhibitory rates of tumor growth were calculated as 100% × (1-average treated tumor size/average control tumor size). **(D)** Antitumor efficacy of BEZ235 and decitabine *in vivo* by tumor weight. Nude mice were killed, and tumors were isolated to weigh each tumor. **(E)** The mRNA levels of PPP2R2B and PTEN were determined by q-PCR in parental and resistant xenografts. **(F)** Protein expression of survival pathway components was determined by immunoblotting analysis in parental and resistant xenografts. **(G)** Protein expression of survival pathway components was determined in parental and resistant xenografts by immunoblotting analysis following treatment with decitabine *in vivo*.

To test whether targeting of methyltransferases could be an effective strategy to overcome BEZ235 resistance *in vivo*, we first constructed refractory mouse models by transplanting the parental and the resistant cells (Figure 7A and 7B). Inhibitory rates of BEZ235 were calculated (Figure 7C). Refractory tumors displayed significant shrinkage after decitabine but not BEZ235 treatment (Figure 7A and 7B). In the acquired resistance model, decitabine plus BEZ235 greatly inhibited tumor growth compared with decitabine alone (*P* = 0.03) (Figure 7A). Similar to the tumor volume, the ability of decitabine to overcome BEZ235 resistance was also observed in terms of the tumor weight (Figure 7D).

To confirm that gene methylation activation of survival pathways induced BEZ235 resistance, the mRNA levels of PTEN and PPP2R2B mRNA were detected. The results showed that their expression decreased in refractory xenograft tumors (Figure 7E). Phosphorylation of AKT, GSK3β, P70 and MYC were elevated in refractory tumors but decreased after decitabine treatment (Figure 7F and 7G). Collectively, these data indicate that inhibition of methyltransferase activity by decitabine sensitized BEZ235-resistant tumors to BEZ235 *in vivo*.

## DISCUSSION

Tumor cells have been shown to develop alternative compensatory pathways to sustain cell proliferation, ultimately leading to drug resistance, such as HER3, IGF-1R, EGFR and insulin receptor pathways [5, 8, 21, 22]. To develop more effective regimens to overcome drug resistance, further dissection of the signaling events that occur in resistant tumors is needed. Identification of the initial factor involved in multiple resistance mechanisms may help researchers to rationally design new combinatorial therapies.

In this study, we demonstrated that DNA methyltransferase is a key modulator that can induce PTEN and PPP2R2B gene hypermethylation, leading to the downregulation of their expression in BEZ235-resistant cancer cells. Reduced PTEN and PPP2R2B expression correlated with activated AKT/mTOR and PDK1/MYC pathways, which can confer considerable BEZ235 resistance in nasopharyngeal carcinoma. Inhibition of methyltransferases in combination with BEZ235 sensitized BEZ235-resistant cells to BEZ235 *in vitro* and *in vivo*.

Due to the substrate diversity of DNMTs, it is not surprising that numerous tumor suppressor genes are hypermethylated in resistant cells [41–46]. We hypothesize that upregulation of DNMT-associated gene hypermethylation may promote the deregulation of PTEN and PPP2R2B that is required for activation of the PI3K/mTOR and PDK1/MYC oncogenic signaling pathways, thus promoting the survival and proliferation of resistant cells. This places DNMTs at a common signaling node for various mechanisms underlying BEZ235 resistance. Thus, we propose that rather than targeting each of the signaling pathways individually, inhibition of this common key node may effectively overcome BEZ235 resistance. This new combination strategy warrants further clinical investigation in patients with tumors carrying a PIK3CA mutation or other factors that activate the PI3K/mTOR pathway.

We tested whether the PDK1 inhibitor GSK2334470 could overcome BEZ235 resistance. In comparison to BEZ235 plus GSK2334470 versus BEZ235 plus decitabine, we observed that resistant cells were more sensitive to decitabine, and the combination with decitabine was more effective compared with the other treatments in overcoming BEZ235 resistance (Figures 2J and 6G). Although the phosphorylation of MYC and P70 was blocked by GSK2334470, the enhanced dephosphorylation of MYC and P70 by decitabine were more pronounced in BEZ235-resistant cells.

In addition to the AKT/mTOR and PDK1/MYC pathways, DNA methyltransferase also modulates many downstream targets, including STAT1, STAT2, STAT3, P21 and P16 [47, 48]. These signaling pathways have diverse roles in regulating tumor cell survival and metastasis. Decitabine affects DNMT enzyme activity rather than its expression [49], which induces genome-wide demethylation to significantly increase the responses of tumor cells to treatment with chemotherapeutic agents such as gemcitabine, temozolomide [50] and cisplatin [40, 46, 51, 52]. This attribute provides a possible application of decitabine in cancer therapy in combination with other drugs. Combinatorial therapy with BEZ235 and decitabine could have a broad impact on tumor progression.

In conclusion, our studies identified DNA methylation as a key convergent point of multiple BEZ235 resistance mechanisms. The combinatorial regimen of the DNA methyltransferase inhibitor plus BEZ235 is effective in overcoming acquired BEZ235 resistance. Our findings may directly improve the outcomes of patients receiving BEZ235 therapy in clinical trials.

## METHODS

### Cell culture and reagents

Human nasopharyngeal carcinoma cells (CNE2, HONE1) were cultured in RPMI 1640 medium supplemented with 10% fetal bovine serum in a humidified atmosphere containing 5% CO2 at 37°C. The approaches to modeled the resistant cell sublines was published before [53]. Acquired BEZ235 resistance were generated as described previously. Briefly, parental cells were continuously exposed to increasing doses of BEZ235 (from 0.05 μmol/L up to 0.4 μmol/L) for 6 to 9 months.

BEZ235, GSK2334470, JQ-1, decitabine and RG108 were purchased from Selleck Chemicals. Human normal IgG was obtained from Roche (Basel, Basel-Stadt, Switzerland). GAPDH antibody was obtained from Boster Biological Technology (Wuhan, China). MTT was purchased from Sigma-Aldrich (St. Louis, Missouri), and phospho-AKT, phospho-MYC, MYC, GSK3α/β antibodies were from Santa Cruz Biotechnology (Indian Gulch, California). All other antibodies were purchased from Cell Signaling Technology (Beverly, Massachusetts). Matrigel was purchased from BD Sciences. The Annexin V-FITC Apoptosis Detection Kit was from Invitrogen Inc. (Carlsbad, California).

### Plasmid construction, retrovirus production and infection

The human PPP2R2B vector was from Origene (RC220271). Lentiviral-based PPP2R2B shRNA vectors and the control shRNA vector and packaging vectors (psPAX2 and pMD2.G) were from GeneChem (Shanghai, China). The PPP2R2B-targeting shRNA sequences used in the lentiviral constructs were as follows: 5′-CCGG GCGGCTACAAATAACCTATATCTCGAGATATAGGT TATTTGTAGCCGCTTTTTG-3′; 5′-AATTCAAAAAGC GGCTACAAATAACCTATATCTCGAGATATAG GTTA TTTGTAGCCGC-3′; and 5′-CCGGCAGCGACTATG AAACCTACATCTCG AGATGTAGGTTTCATAGTC GCTGTTTTTG-3′; 5′-AATTCAAAAACAGCGAC TA TGAAACCTACATCTCGAGATGTAGGTTTCATAGTC GCTG-3′. For lentiviral production, the lentiviral expression vector was co-transfected with third-generation lentivirus packing vectors into 293T cells using Lipofectamine Reagent (Invitrogen, Carlsbad, California). At 48 to 72 h after transfection, the breast cancer cell lines were stably infected with viral particles.

### Gene expression profiling and quantitative RT-PCR analysis

Total RNA was extracted from the cell lines using TRIzol (Invitrogen) and purified with the RNeasy Mini Kit (Qiagen). Reverse transcription was performed using an RNA Amplification kit (Fermentas). qPCR was performed using a BioRad CXF96 Sequence Detection System (Applied Biosystems) with SyBR Green Master Mix (Roche). Three independent samples, each in triplicate, were analyzed for each qPCR condition. The samples were normalized to the levels of GAPDH mRNA. The PCR primers are described below. The primers used for the quantitative RT-PCR analysis were as follows:

PPP2R2B (human) F: 5′-GCGTGATAAGAGG CCAGAAG-3′, R: 5′-TGTGTGCG TTGGCAAATACT-3′;

PTEN (human) F: 5′-ACCAGGACCAGAGGAAA CCT-3′, R: 5′-GCTAGC CTCTGGATTTGACG-3′

DNMT1 (human) F: 5′-GAGCTACCACGCAGAC ATCA-3′

R: 5′-CGAGGAAGTAGAAGCGGTTG-3′

DNMT2 (human) F: 5′-TGGAAACCAAGGCAG TTTTC-3′

R: 5′-GCAAGGGTCTAGAGGGGAAC-3′

DNMT3A (human) F: 5′-CCGGAACATTGAGG ACATCT-3′

R: 5′-CAGCAGATGGTGCAGTAGGA-3′

DNMT3B (human) F: 5′-CACGCAACCAGAG AACAAGA-3′

R: 5′-GGCAACATCTGAAGCCATTT-3′

DNMT3L (human) F: 5′-CTCTCAAGCTCCG TTTCACC-3′

R: 5′-GTACAGGAAGAGGGCATCCA-3′

GAPDH (human) F: 5′-AAGCCTGCCGGTGA CTAAC-3′

R: 5′-GTTAAAAGCAGCCCTGGTGAC-3

PPP2R2B (mouse) F: 5′-ATCCAAACCCATTCC TTTCC-3′

R: 5′-GAAACCCAGCAGCAGACTTC-3′

PTEN (mouse) F: 5′-ACAATTCCCAGTCAGA GGCG-3′

R: 5′-CCTTTAGCTGGCAGACCACA-3′

GAPDH (mouse) F: 5′-AACTTTGGCATTGTGG AAGG-3′

R: 5′-ACACATTGGGGGTAGGAACA-3

### RNA Interference

List of siRNA sequences

MYC-A F: 5′-GUGCAGCCGUAUUUCUACUTT-3′

R: 5′-AGUAGAAAUACGGCUGCACTT-3′

MYC-B F: 5′-CCAAGGUAGUUAUCCUUAATT-3′

R: 5′-UUAAGGAUAACUACCUUGGTT-3′

S6K-1 F: 5′-GCACCUGCGUAUGAAUCUATT-3′

R: 5′-UAGAUUCAUACGCAGGUGCTT-3′

S6K-2 F: 5′-GUGCCAAUCAGGUCUUUCUTT-3′

R: 5′-AGAAAGACCUGAUUGGCACTT-3′

PDK1–1 F: 5′-CAAAGUUCUGAAAGGUG AAAU-3′

R: 5′-AUUUCACCUUUCAGAACUUUG-3′

PDK1–2 F: 5′-GCAGCAACAUAGAGCAGU ACA-3′

R: 5′-UGUACUGCUCUAUGUUGCUGC-3′

PDK1–1 F: 5′-CAAAGUUCUGAAAGGUG AAAU-3′

R: 5′-AUUUCACCUUUCAGAACUUUG-3′

PDK1–2 F: 5′-GCAGCAACAUAGAGCAGU ACA-3′

R: 5′-UGUACUGCUCUAUGUUGCUGC-3′

PIM1–1 F: 5′-TGGAAACCAAGGCAGTTTTC-3′

R: 5′-GCAAGGGTCTAGAGGGGAAC-3′

PIM1–2 F: 5′-CCGACAGUUUCGUCCUGA UTT-3′

R: 5′-AGGAAUAUCUCCACACACCTT-3′

PTEN-1 F: 5′-CCACCACAGCUAGAACUU ATT-3′

R: 5′-UAAGUUCUAGCUGUGGUGGTT-3′

PTEN-2 F: 5′-CCAGUCAGAGGCGCUAUG UTT-3′

R: 5′-ACAUAGCGCCUCUGACUGGTT-3′

NC F: 5′-UUCUCCGAACGUGUCACGUTT-3′

R: 5′-ACGUGACACGUUCGGAGAATT-3′

Cells were transfected with 100 nM siRNA duplexes using

Lipofectamine RNAiMAX (Invitrogen) according to the manufacturer's instructions.

### Cell cycle assay

The cell cycle distribution was determined by fluorescence activated cell sorting (FACS) (Beckman Coulter, FC500MCL). CNE2 and CNE2/235 cells were trypsinized and fixed in 70% ethanol at −20°C overnight. Cells were resuspended in PBS containing 40 μg/ml propidium iodide and 100 μg/ml RNase A (Sigma). After incubation for 3 min at 37°C, the cells were characterized. Data were acquired using a BD LSRII apparatus and were analyzed using Cytomics RXP Analysis software (Beckman Coulter, Brea, CA, USA).

### Western blot analysis and immunoprecipitation

Cells were collected on ice and washed with PBS before being lysed in cell lysis buffer (Cell Signaling Technology) containing Complete Protease Inhibitor Cocktail (Roche Applied Sciences). The cell lysate was collected after centrifugation (12,000 rpms, 15 minutes). The protein concentration was determined using the Pierce BCA protein assay kit (Thermo Scientific Pierce, Rockford, IL, USA), and the proteins were separated electrophoretically in SDS-polyacrylamide gels and transferred to PVDF membranes (Roche). After blocking with 5% milk in TBST containing 0.05% Tween 20 (PBST) for 2 h, the membrane was probed with various primary antibodies overnight at 4°C, incubated with HRP-conjugated secondary antibodies for at least 2 h at room temperature, washed with TBST and visualized using enhanced chemiluminescence reagent according to the manufacturer's instructions (Thermo Fisher Scientific Inc). Immunoreactivity was detected using Amersham ECL Prime Western Blot Detection Reagent (GE Healthcare, Fairfield, CT, USA) according to the manufacturer's instructions. For the co-immunoprecipitation experiments, cells were lysed in ice-cold E1A lysis buffer (250 mM NaCl, 50 mM HEPES [pH 7.5], 0.1% NP-40, 5 mM EDTA, Roche protease inhibitor cocktail). The total cell lysate was incubated with primary antibodies overnight at 4°C. The immunocomplex was precipitated using protein A or G agarose for another 4 h at 4°C. Immunoprecipitates were washed 3 times with E1A lysis buffer and denatured by the addition of 20 ml 1XSDS sample buffer and boiling for 5 min. They were then separated by SDS-PAGE for Western blot analysis.

### *In vivo* studies

Female athymic BALB/c nude mice (4–6 weeks old) were purchased from Hunan Silaikejingda Laboratory Animal Technology Co. Ltd (Changsha, China). Mice were implanted subcutaneously in the flank with 1 × 10^6^ CNE2 cells. Tumors reached a size of ~50 mm^3^, and serial establishment of refractory xenografts was performed using xenograft tumors formed from CNE2 cells. In brief, subcutaneous tumors were excised and minced prior to subcutaneous embedding into nude mice. When the tumors reached ~200 mm^3^, BEZ235 was administered po at 25 mg/kg per day for 4 weeks. For two BEZ235 treatment cycles, refractory xenografts were established by a repeated embedding. When the tumors reached ~200 mm^3^, the mice were divided into four groups (4 mice per group). The BEZ235 was administered po at 25 mg/kg per day, and decitabine was administered via intraperitoneal injection at 0.03 mg/kg three times per week. Tumor progress was monitored based on the whole body weight and tumor size every 3–4 days following injection. Tumor growth was followed for 4 weeks. Animals were killed, and tumors were resected and preserved at −80°C for Western blot and quantitative RT-PCR analyses.

### Statistical analysis

Data are presented as the mean ± SEM unless otherwise stated. Student's *t* test was used to compare two groups for statistical significance analysis.

## SUPPLEMENTAL FIGUREs AND TABLES


