# Supplementary material for: Inhibition of DNA methyltransferase as a novel therapeutic strategy to overcome acquired resistance to dual PI3K/mTOR inhibitors
Source: Oncotarget. 2015 Feb 11;6(7):5134–46. doi: 10.18632/oncotarget.3016 (PMC4467138; doi:10.18632/oncotarget.3016)
Supplement: Supplementary file 1 [file oncotarget-06-5134-s001.pdf]

SUPPLEMENTAL FIGURES AND TABLES

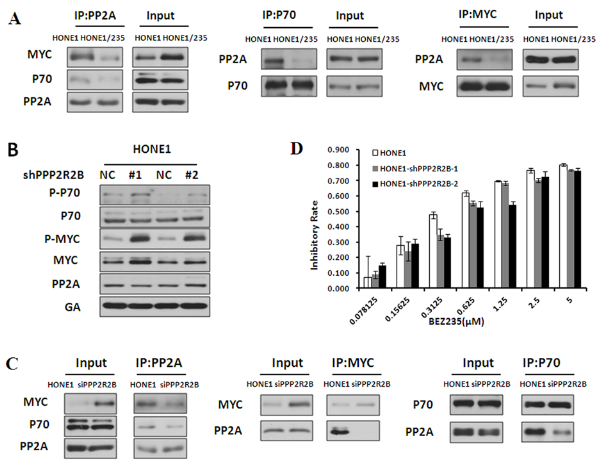

Supplementary Figure S1: (A) The interactions between MYC and PP2A and between P70 and PP2A were detected by immunoprecipitation in HONE1 and HONE1/235 cell lines. **(B)** Effect of PPP2R2B knockdown with shRNA on MYC and P70 phosphorylation in HONE1 cell line. **(C)** Effect of PPP2R2B knockdown on the interactions between MYC and PP2A and between P70 and PP2A in the HONE1 cell line based on immunoprecipitation. **(D)** The sensitivity of HONE1 to BEZ235 after PPP2R2B knockdown by PPP2R2B shRNA using the MTT assay.

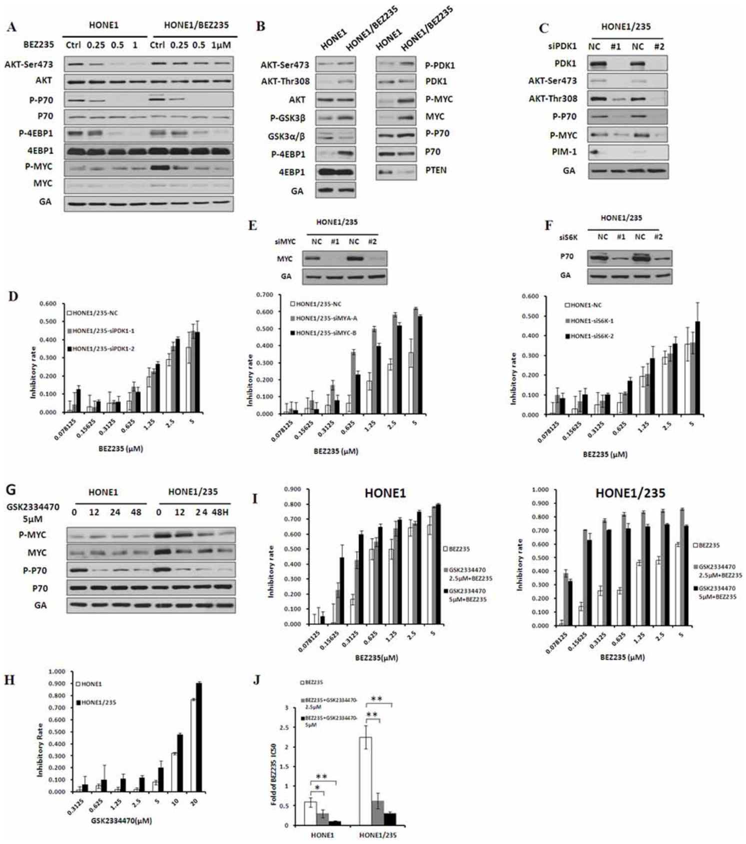

Supplementary Figure S2: (A) Effect of BEZ235 on the PI3K/AKT/mTOR and PDK1/MYC survival pathway in HONE1 and HONE1/235 cells by immunoblotting analysis. **(B)** Differential activation of the PI3K/AKT/mTOR and PDK1/MYC survival pathways in HONE1 and HONE1/235 by immunoblotting analysis. **(C)** Effect of PDK1 knockdown on P-AKT, P-P70, P-Myc, and PIM1 expression in HONE1/235 cells. **(D)** Sensitivity to BEZ235 in HONE1/235 cells after PDK1 knockdown using the MTT assay. **(E)** Effect of MYC knockdown on the sensitivity to BEZ235 in HONE1/235 cells. **(F)** Effect of P70(S6K) knockdown on the sensitivity to BEZ235 in HONE1/235 cells. **(G)** Effect of the PDK1 inhibitor GSK2334470 on MYC and P70 phosphorylation in HONE1/235 cell line by immunoblotting analysis. **(H)** Sensitivity to GSK2334470 in HONE1/235 cells after treatment with GSK2334470 for 5 d using the MTT assay. **(I)** Inhibitory effect of GSK2334470 in combination with BEZ235 on cell proliferation in HONE1/235 cells using the MTT assay. **(J)** IC50 values of BEZ235 with or without GSK2334470 in HONE1 and HONE1/235 cell lines. The data shown are representative of 3 individual experiments.

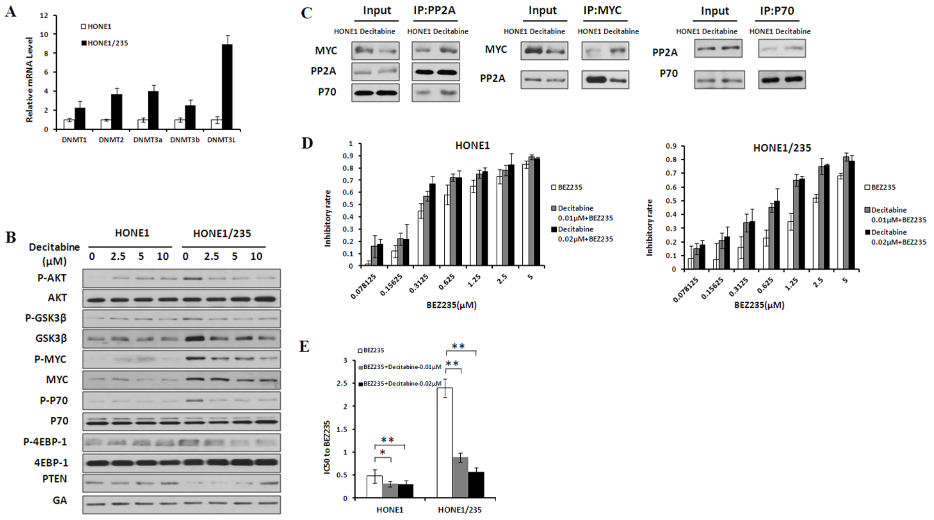

Supplementary Figure S3: **(A)** The mRNA levels of DNA methyltransferases were analyzed in HONE1 and HONE1/235 cell lines by q-PCR. **(B)** Effects of decitabine on the expression of survival pathway proteins at the indicated concentrations. **(C)** Effects of decitabine on the binding of MYC and PP2A and of P70 and PP2A in the HONE1/235 cell line. Cells were treated with 1 μM decitabine for 48 h. Immunoprecipitation was conducted. **(D)** Inhibitory effect of decitabine and BEZ235 on cell proliferation in HONE1 and HONE1/235 cell lines. Cells were treated for 5 d at the indicated concentrations. The MTT assay was conducted. **(E)** The IC50 values of BEZ235 with or without decitabine were determined in HONE1 and HONE1/235 cell lines using the MTT assay. The data shown are representative of 3 individual experiments.

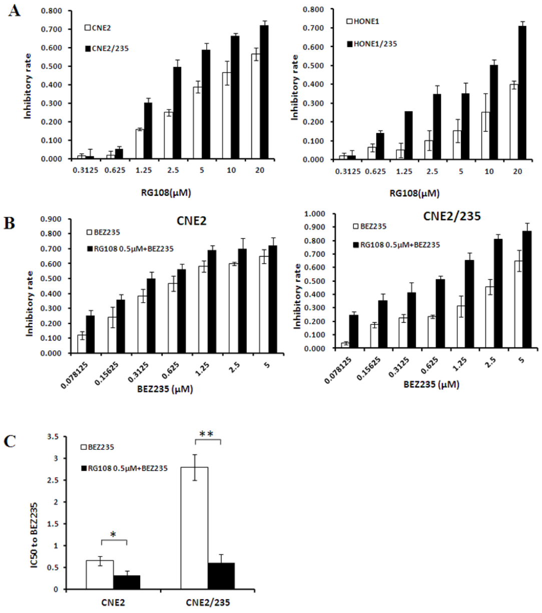

Supplementary Figure S4: **(A)** Effect of the DNA methylation inhibitor RG108 on cell proliferation in the parental and resistant cell lines using the MTT assay. **(B)** Inhibitory effect of RG108 and BEZ235 on cell proliferation in BEZ235-resistant cell lines. Cells were treated for 5 d at the indicated concentrations. The MTT assay was conducted. **(C)** The IC50 values of BEZ235 with or without RG108 were determined in parental and resistant cell lines using the MTT assay. The data shown are representative of 3 individual experiments.

Supplementary Table S1: Oncomutation panel mutation list report

Sample	Gene	Assay	Mutation	Allele	WT Frequency	Mutation Frequency	Z-Score	Confidence
CNE1	HRAS	HRAS_3	G13R	G	0.808	0.193	10	High
CNE1	HRAS	HRAS_3	G13R	G	0.81	0.19		
CNE2	HRAS	HRAS_3	G13R	G	0.753	0.218	10	High
CNE2	HRAS	HRAS_3	G13R	G	0.75	0.22		
CNE2	PIK3CA	PIK3CA_9	H1047R	G	0.363	0.637	10	High
CNE2	PIK3CA	PIK3CA_9	H1047R	G	0.45	0.55		
HONE1	HRAS	HRAS_3	G13R	G	0.796	0.204	7.98	High
HONE1	HRAS	HRAS_3	G13R	G	0.8	0.2		
HONE1	PIK3CA	PIK3CA_9	H1047R	G	0.497	0.503	10	High
HONE1	PIK3CA	PIK3CA_9	H1047R	G	0.5	0.5		
SUNE1	PIK3CA	PIK3CA_9	H1047R	G	0.484	0.516	10	High
SUNE1	PIK3CA	PIK3CA_9	H1047R	G	0.48	0.52		

Supplementary Table S2: PPP2R2B and PTEN gene methylation were increased in BEZ235 resistance cell line

	CNE2-235/CNE2 DiffScore	CNE2-235/CNE2 Delta.Beta	Gene region
PPP2R2B	374.3439	0.560642	Island
PTEN	65.58192	0.2575	Body

DiffScore > 13 and Delta.Beta >0.17 means hypermethylation.
